# The future impact of zero-dose children in inaccessible conflict-affected areas of Somalia: aligned with the immunization agenda 2030

**DOI:** 10.1186/s41182-025-00833-2

**Published:** 2025-11-12

**Authors:** Saadaq Adan Hussein, Marian Muse Osman, Mohamed Mohamoud Hassan, Yahye Sheikh Abdulle Hassan, Abdirahman Aden Hussein, Abdinur Hussein Mohamed, Rage Adem, Mohamed MAli Fuje, Khadar Hussein Mohamud, Ayan Nur Ali, Abdirahman Moallim Ibrahim, AbdulJalil Abdullahi Ali

**Affiliations:** 1 Department of School of Postgraduate Studies, Benadir University, Mogadishu, Somalia; 2 Department of Social and Human Capital Development Pillar, Office of the Prime Minister, Mogadishu, Somalia; 3 Department of Research and Policy Development, SOR Institute: Somalia Social Research, Mogadishu, Somalia; 4 Department Benadir Institute for Research and Development, Benadir University, Mogadishu, Somalia; 5 Department Research, Somali National Institute of Health, Mogadishu, Somalia; 6 Department Rector at, Benadir University, Mogadishu, Somalia; 7 Department Medicine and Surgery, Jamhuriya University of Science and Technology, Mogadishu, Somalia; 8 Department Director Innovation Hub, Benadir University, Mogadishu, Somalia; 9 Department Executive of Somali Development Research Institute (SODRI), Mogadishu, Somalia; 10 Department of Hemodialysis at Mogadishu, Somali Türkiye Training and Research Hospital, Mogadishu, Somalia; 11 Departments Faculty of Medicine and Surgery, Jazeera University, Mogadishu, Somalia

**Keywords:** Zero-dose, Children, Unvaccinated, Somalia, Inaccessible, Conflict-affected, Unliberated areas, Immunization, Conflict, Zones

## Abstract

**Background:**

The Immunization Agenda 2030 (IA2030), led by WHO and partners, targets the global challenge of zero-dose children, who face higher risks of vaccine-preventable diseases. Globally, 18 million children remain zero-dose, with over half in conflict or humanitarian settings. In Somalia, about 60% of children are zero-dose, and during the 2022–2024 drought, over 70,000 deaths occurred, with nearly 40% among children under five. This review explores the burden, determinants, and geographic distribution of zero-dose children in Somalia's conflict-affected regions.

**Methods:**

This narrative review followed SANRA guidance. We searched PubMed, Scopus, Web of Science, Google Scholar, and key institutional sites (WHO, UNICEF, ReliefWeb, MoH Somalia, NGOs) for English-language literature (1990–July 31, 2025). From 197 records were identified, 82 new studies were included, resulting in a total of 279 studies after de-duplication and two-reviewer screening. Evidence was synthesized thematically and aligned to Immunization Agenda 2030 (IA2030) priorities.

**Results:**

Zero-dose hotspots are concentrated in rural, nomadic, and conflict-affected zones, with Lower Juba reaching a peak of 62%. Key challenges include insecurity, limited access, disrupted supply chains, workforce shortages, and demand-side barriers like mistrust and misinformation. Humanitarian efforts are frequently hindered by checkpoints, blockades, and security concerns. From 2000 to 2024, Somalia's routine immunization program showed significant progress, with MCV-1 coverage rising from 50 to 71%, and MCV-2 from 5 to 55%, as per the WHO/UNICEF WUENIC data for the African region.

**Conclusion:**

Zero-dose children in inaccessible Somali districts are a pressing equity and health-security challenge. Sustaining recent national gains while fulfilling Immunization Agenda 2030 (IA2030)’s “leave no one behind” requires tailored outreach to remote communities, strengthened surveillance and e-registries for defaulter tracing, resilient cold-chain and WASH linkages, empowered community health workers (especially women), negotiated humanitarian access, and a progressive domestic co-financing roadmap alongside partner support.

## Introduction

The Immunization Agenda 2030 (IA2030) is a global strategy led by WHO and its partners [1], aiming to ensure universal vaccine access and prevent vaccine-preventable diseases while reducing mortality, strengthening health systems, and promoting equitable coverage guided by six strategic priorities and four core principles and endorsed by WHO Member States in November 2020, IA2030 envisions a world where vaccines protect health universally, regardless of background [2].

Zero-dose children those who have never received any routine immunizations—or children aged 12–23 months received no doses of the four basic routine vaccines (BCG, Polio, DPT, Measles) [3–5] and face markedly higher risks of morbidity and mortality from vaccine-preventable diseases [6], Routine childhood vaccination is pivotal to reducing under-five deaths globally by protecting against diphtheria, pertussis, tetanus, measles, and poliomyelitis [7, 8].Worldwide, an estimated 18 million children remain zero-dose, with more than half living in conflict-affected or other humanitarian settings [9]. Zero-dose hotspots are concentrated in areas affected by conflict, nomadic migration, and displacement. Weak tracking of internally displaced populations (IDPs) and fragile data systems hinder targeted programming and monitoring. Community mistrust—exacerbated by misinformation and limited engagement—further depresses vaccine demand and confidence [10].

In Somalia, the situation is especially acute: approximately 60% of children have never received a single vaccine dose [11], predominantly in districts where insecurity and poor infrastructure restrict access to health services, and these alarming figures underscore that more than three out of five young children in Somalia are “zero-dose,” a term defined by WHO/IA2030 as having not received even the first routine vaccine dose [12]. Decades of conflict, displacement, and under-funding have splintered immunization programs and eroded health-system capacity [5, 13].During the 2022–2024 drought, more than 70,000 deaths occurred; nearly 40% were among children under five [14].

Since state collapse in 1991, immunization gaps have been driven by insecurity, displacement, disrupted supply chains, and weak outreach [11, 12]. Conflict-related access constraints leave many children unprotected, with persistently low coverage in conflict zones [15]. By 2020, National coverage for routine immunization (BCG, Polio (OPV, IPV), Pentavalent (Penta), and Measles) [16] reached roughly 50–60%. In 2025, Somalia reports a strong rise in child immunization: an estimated 70% of children are fully vaccinated—up 28% points since 2012—while the number of zero-dose children has fallen, reflecting coordinated efforts by the Federal Government, WHO Somalia, UNICEF, Gavi, and partners [17]. Nevertheless, large areas under militant control remain inaccessible [15, 18, 19]. The health system continues to struggle with non-functional facilities, disrupted supply chains, and limited humanitarian access, especially in territories controlled by non-state armed groups [5, 20, 21], Somalia’s EPI is heavily donor-funded—government covered only 0.3% of routine vaccine costs in 2014—with a substantial financing gap reported [22].

**Fig. 1:**
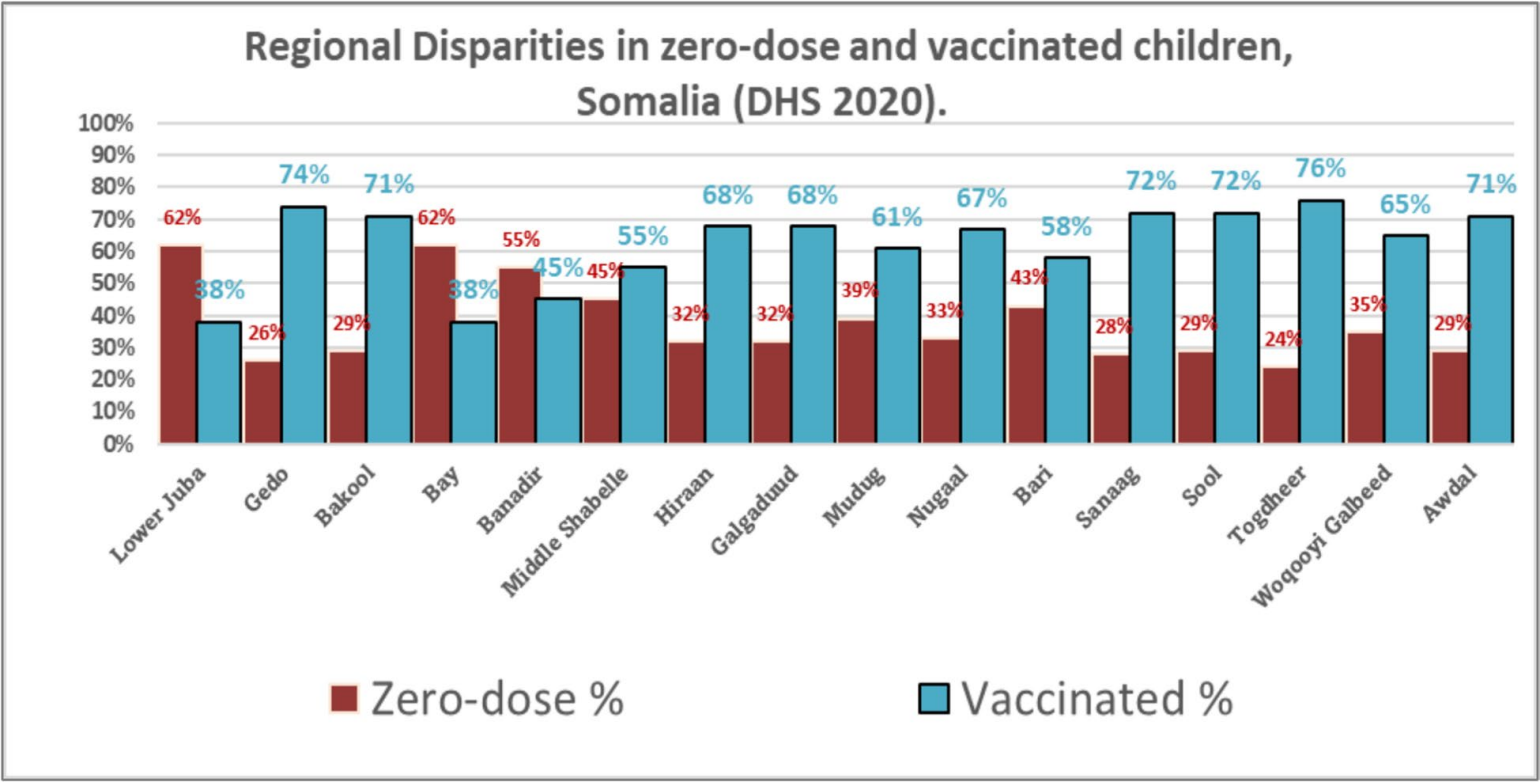
Regional disparities in zero-dose and vaccinated children, Somalia (DHS 2020) [25]

Looking ahead, aid cuts threaten vaccines, staffing, and outreach in many low-income countries after 2025[23]. While many children receive routine vaccines, coverage remains far lower in insecure districts [24] and zero-dose rates reportedly peak at 62% in conflict-affected areas such as Lower Juba [25] (Fig. 1). More than 70% of the population lacks reliable access to care; polio and measles persist in underserved regions [26]. These settings also face deficits in WASH and functional primary health care, compounding public-health risks [21].Persistent service gaps heighten morbidity, deepen inequities, and demand urgent, tailored outreach to remote Somali communities [27]. Addressing these inequities is essential to achieving the Immunization Agenda 2030 and the Sustainable Development Goals’ pledge to “leave no one behind [28].

This narrative review addresses critical gaps data on zero-dose children in Somalia’s conflict-affected regions by assessing their burden, key determinants, and geographic distribution. We align these insights with Immunization Agenda 2030 (IA2030) targets to inform equity-driven immunization strategies so that no child is left behind in fragile and inaccessible settings.

## Methods: Study area and study design

This narrative review employed a SANRA (Scale for the Assessment of Narrative Review Articles) [29] methodology to synthesize current evidence on the burden, predictors, and implications of zero-dose children in inaccessible and conflict-affected regions of Somalia. Somalia, a low-income, conflict-fragmented federal republic in the Horn of Africa, has an estimated population of 18.1 million. The health system is bifurcated between the Federal Ministry of Health and six state-level ministries, with service delivery concentrated in urban and rural areas, However, vast rural and militant-controlled areas particularly in south-central Somalia remain largely unreachable by vaccination campaigns, harboring disproportionately high numbers of zero-dose children. These regions suffer from chronic underinvestment in health infrastructure, low health workforce density, and heavy dependence on donor funding, which accounts for approximately 50% of total health expenditure. Surveillance systems, though supported by frameworks like the Integrated Disease Surveillance and Response (IDSR), remain incomplete, especially in districts with high numbers of internally displaced persons (IDPs), estimated at over 2.6 million. Compounding these structural barriers are recurrent disease outbreaks—such as measles, cholera, and polio driven by climatic shocks, drought-induced water scarcity, and flood-related WASH failures [12, 30].

## Literature review strategy and data extraction process

This narrative review synthesizes findings from peer-reviewed articles, government reports, and grey literature on the future impact of zero-dose children in inaccessible, conflict-affected areas of Somalia. The review aimed to addresses critical gaps data on zero-dose children in Somalia’s conflict-affected regions by assessing their burden, key determinants, and geographic distribution, with a specific focus on Somalia. A systematic search was conducted across four academic databases—PubMed, Scopus, Web of Science, and Google Scholar—along with institutional websites of key organizations such as WHO, UNICEF, Relief Web, the Ministry of Health Somalia, and relevant implementing NGOs.

**Fig. 2:**
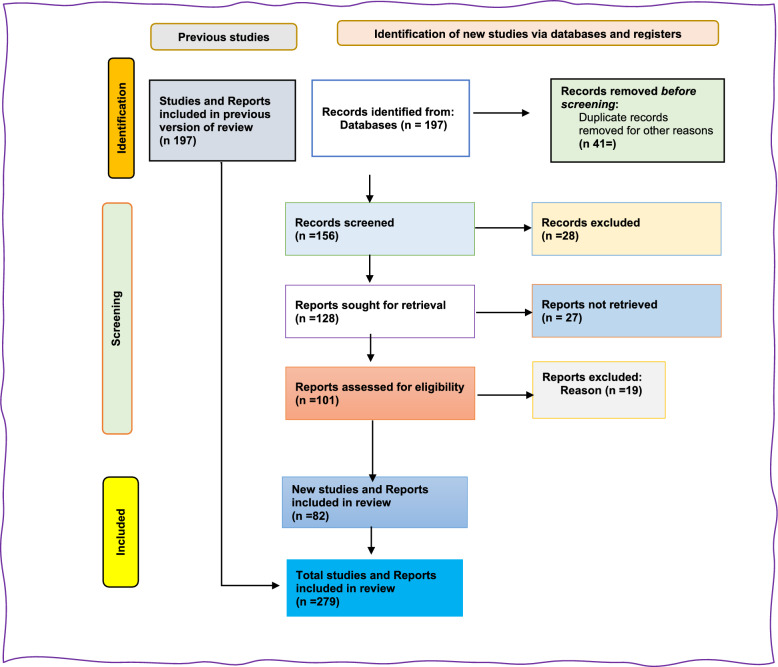
PRISMA 2020 Flow Diagram

The search covered literature published between 1990 and 2024, with the final retrieval completed on Between January to July 31, 2025. Search terms included combinations of the following keywords: ("Immunization Agenda 2030″ OR IA2030) AND ("immunization equity" OR "Immunization equity") AND (Somalia OR Somali) AND ("zero-dose child" OR unvaccinated*) AND (in access* OR "conflict-affected" OR "conflict zone*" OR "unliberated area*”) *. Only English-language sources were included due to translation limitations. From 197 records were identified, 41 removed before screening, and 156 were screened. After seeking 128 reports for retrieval and assessing 101 for eligibility, 82 new studies were included, resulting in a total of 279 studies being included in the final review (Fig. 2).

### Inclusion criteria

Published between January 1990 and March 2025 and Focus on Somalia or Horn of Africa in fragile/conflict-affected settings English only.

### Exclusion criteria

- Non-English publications, and Editorials, commentaries, or opinion pieces without empirical data
- Studies unrelated to immunization or zero-dose children in the specified region

### Data organization and thematic synthesis

The data collected from 112 eligible sources PubMed, Scopus, Web of Science, and Google Scholar—along with institutional websites of key organizations such as WHO, UNICEF, Relief Web, MOH, and relevant implementing NGOs were systematically organized and synthesized using an inductive thematic analysis to explore the determinants, barriers, and implications of zero-dose children in conflict-affected and inaccessible regions of Somalia. The analysis was conducted using NVivo 12 and followed Braun and Clarke’s six-step framework, including familiarization with the data, generating initial codes, searching for themes, reviewing themes, defining and naming themes, and writing the report. Two independent reviewers conducted the screening and data extraction processes, resolving any discrepancies through consensus. Six key themes emerged from the analysis included 1. Epidemiological Landscape, 2. Health Implications, 3. Immunization Strategies, 4. Policy and Governance, 5. Community Engagement, 6. Future Directions and Recommendations. This thematic synthesis offers a multidimensional view of the zero-dose challenge in Somalia and provides a structured foundation for developing targeted, equity-oriented immunization policies that are resilient to both political and environmental shocks.

## Result: Thematic analysis for the narrative review

### Epidemiological Landscape

#### Magnitude and Spatial Distribution

Somalia faces a profound immunization gap, with reports indicating that approximately 60% of children aged 12–23 months received no doses of the four basic routine vaccines (BCG, Polio, DPT, Measles) [3, 4, 11]. Another national study found that 65% of under-five children remained entirely unvaccinated [31]. Zero-dose children are mostly found in rural, nomadic, and conflict-affected areas. SHDS 2020 data shows zero-dose children live in these settings, with an adjusted odds ratio of 1.52 times higher in rural/nomadic areas than urban ones [11].

**Fig. 3:**
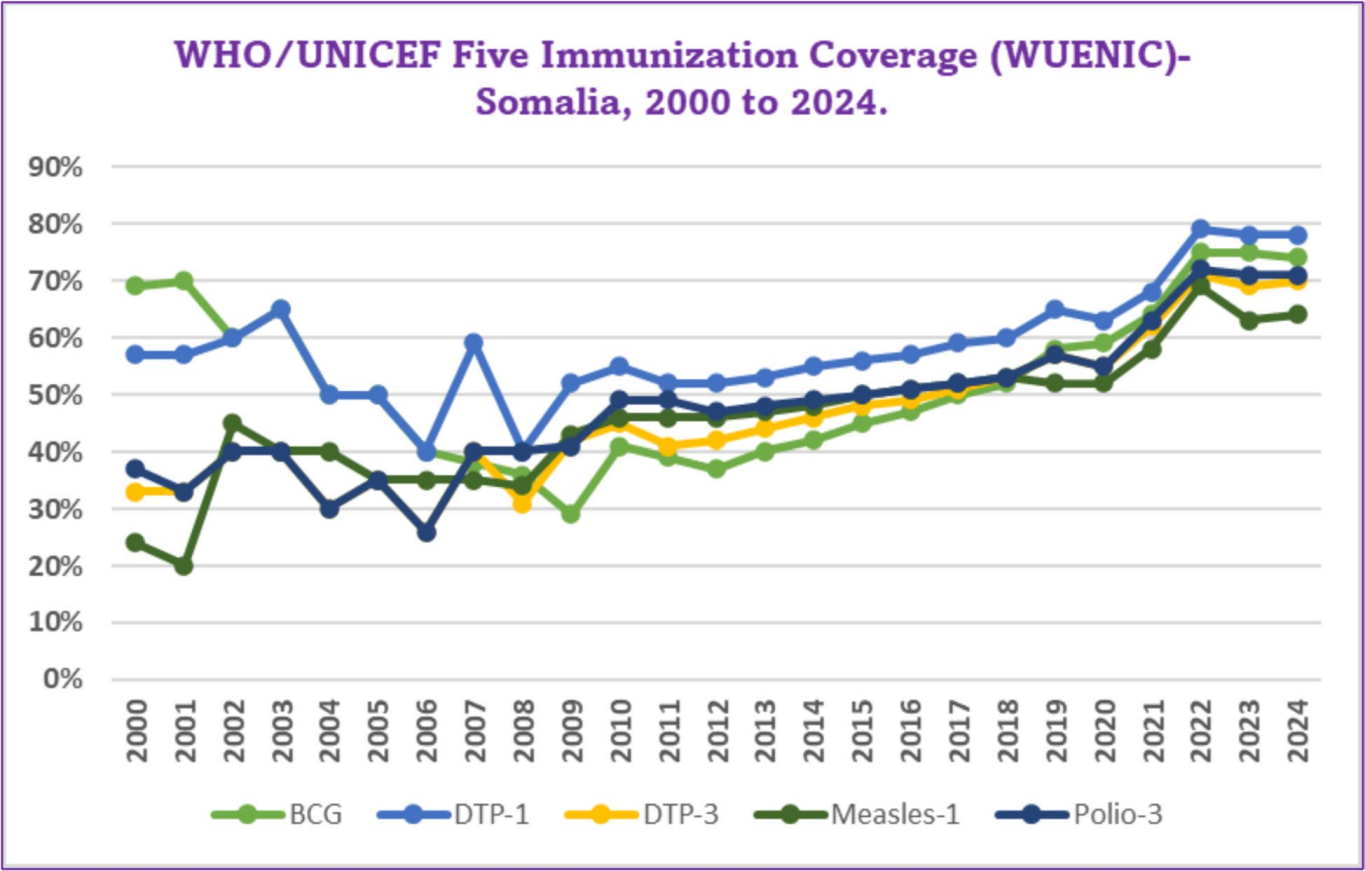
WHO/UNICEF Five Immunization Coverage (WUECIN)-Somalia, 2000–2024

Somalia’s routine immunization programme has moved from a low-coverage, stagnating platform in the early 2000s to one showing sustained, broad-based gains by 2024. At the turn of the millennium, scarcely one child in three completed the DTP series (33%) and fewer than a quarter received the first measles dose (24%), while coverage for the third polio dose stood at 37%. Progress was slow and uneven through 2010, marked by brief setbacks linked to conflict and population displacement, BCG fell to 40% in 2006 and Polio-3 to 26%. From 2011 onward, however, the curve ticks steadily upward: DTP-3 climbs past 50% in 2017, Polio-3 follows in 2018, and Measles-1 edges above 50% in 2015 before accelerating after 2020. All antigens leap by ~ 10 percentage points in just two years: BCG rises from 64% (2021) to 75% (2022); DTP-1 from 68 to 79%; DTP-3 from 62 to 71%; Measles-1 from 58 to 69%; and Polio-3 from 63 to 72% (Fig. 3) [30].

**Fig. 4:**
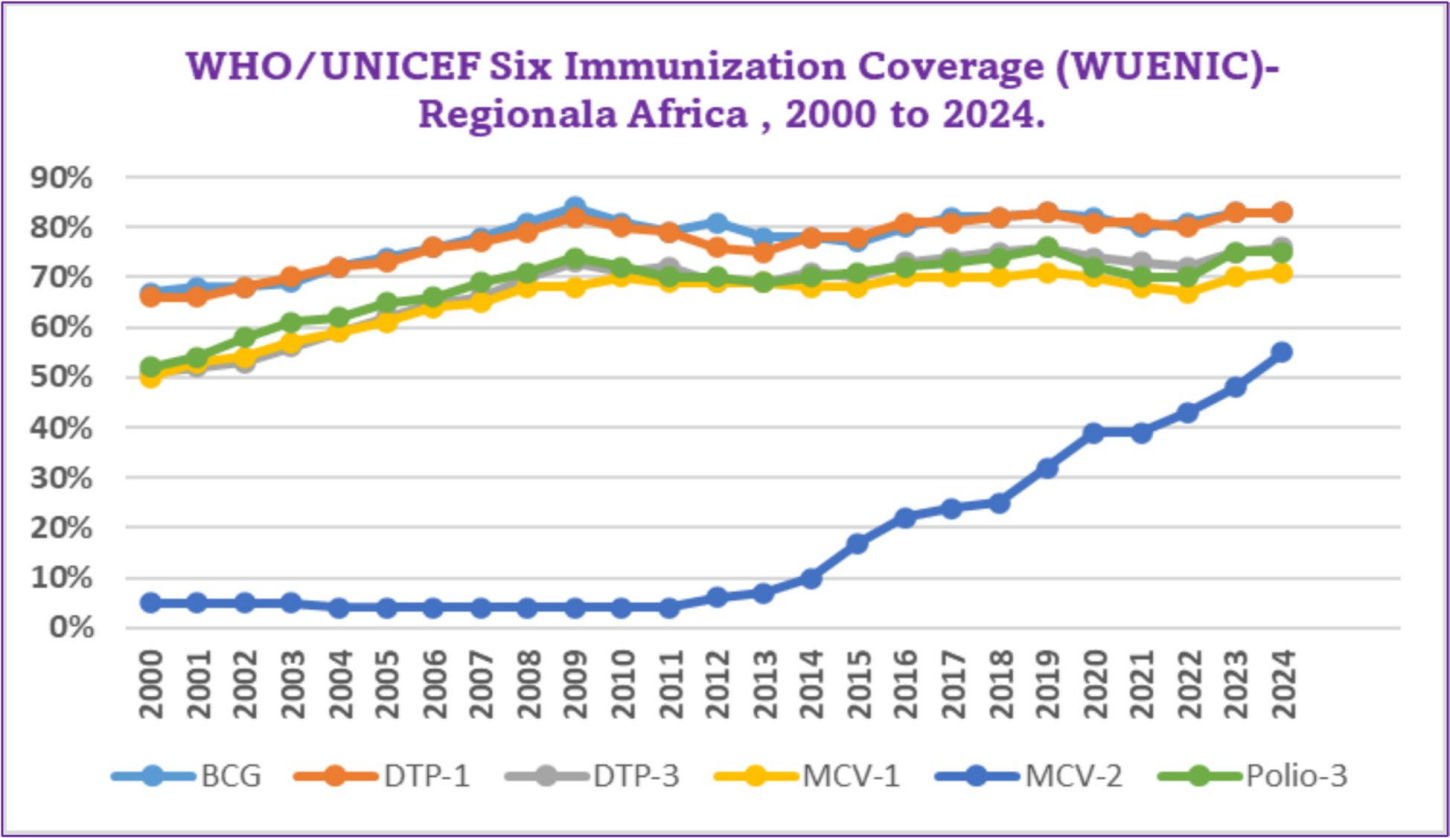
WHO/UNICEF Five Immunization Coverage (WUECIN)-Somalia, 2000–2024

Data from the WHO/UNICEF Six Immunization Coverage (WUENIC) for the African region between 2000 and 2024 reveals consistent improvements in immunization rates across multiple vaccines. BCG coverage increased from 67% in 2000 to 83% by 2024. DTP-1 coverage rose from 66% in 2000 to 83% in 2024, while Polio-3 coverage advanced from 52% in 2000 to 75% in 2024. Significant gains were also observed in measles vaccines, with MCV-1 climbing from 50% in 2000 to 71% in 2024, and MCV-2 increasing from 5% in 2000 to 55% in 2024. DTP-3 coverage steadily improved from 51% in 2000 to 76% in 2024. These trends indicate overall progress in African immunization, highlighting the importance of sustained vaccination campaigns, though challenges persist in certain areas (Fig. 4) [32].

The Epidemiological Spatial Distribution map provides a visual representation of zero-dose immunization rates across various regions of Somalia, highlighting regional disparities that are essential for prioritizing immunization efforts. These disparities often align with conflict-affected areas, particularly in South-Central Somalia, where insecurity has limited survey coverage. The regions most affected by conflict include Bay, Lower Juba, Middle Shabelle, Mudug, and Galgaduud, which have the highest zero-dose rates. These areas remain challenging due to ongoing political tensions and instability. Additionally, regions such as Sanaag and Awdal are also impacted by political unrest, contributing to their low immunization coverage.

**Fig. 5:**
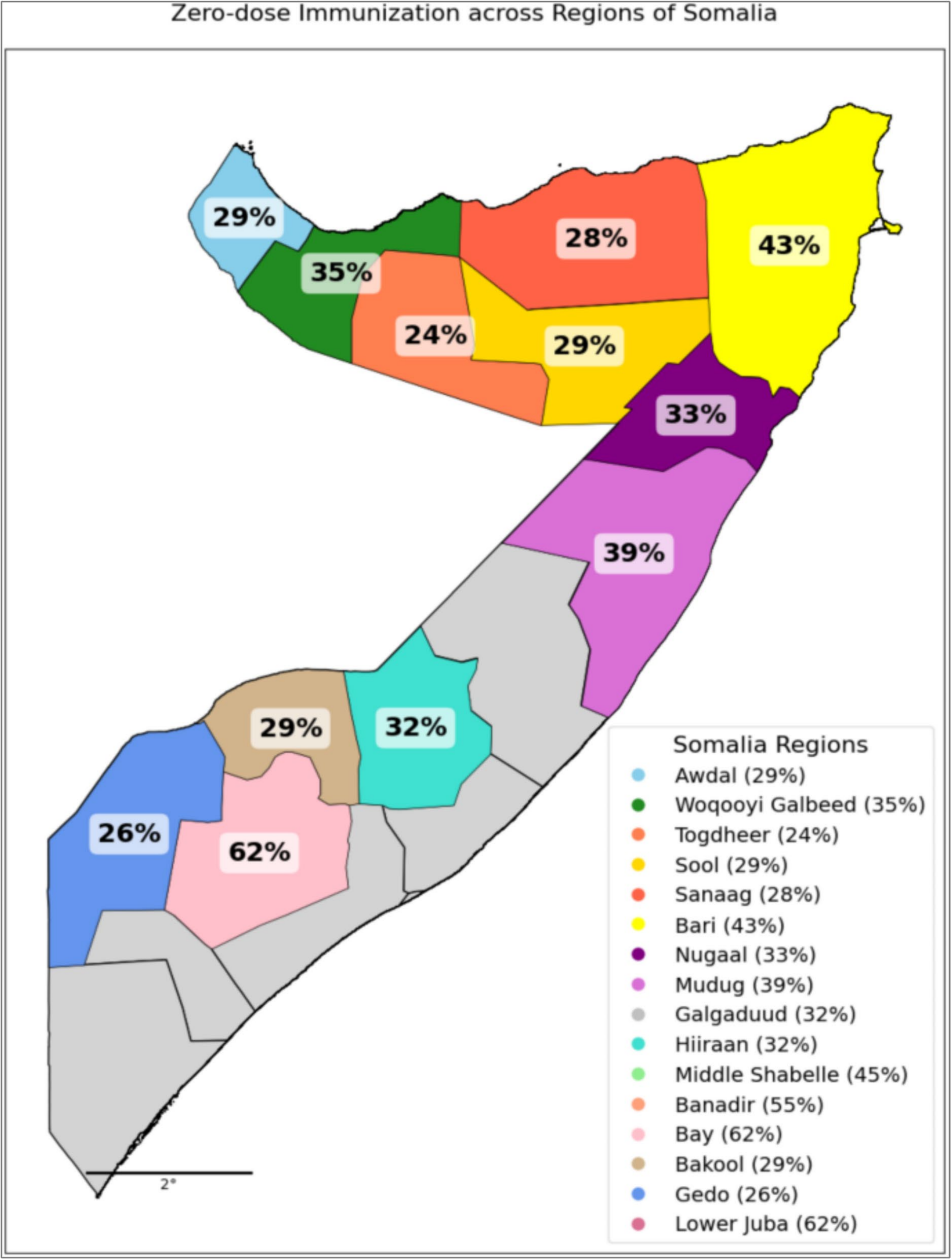
Zero-dose Immunization Rates Across Regions of Somalia

The map reflects varying immunization rates, which are critical for understanding vaccination gaps and targeting interventions effectively. Bay and Lower Juba have the highest immunization rates at 62%, followed by Banadir and Middle Shabelle with 55% and 45%, respectively. Woqooyi Galbeed and Mudug show 35% and 39%, while Togdheer has the lowest at 24%. Other regions like Sool, Sanaag, and Awdal exhibit moderate coverage at 29%, 28%, and 29%, respectively. Addressing these disparities, particularly in conflict and politically unstable areas, is crucial for reducing the number of zero-dose children in Somalia. (Fig. 5) [25].

The prevalence of zero-dose vaccination is disproportionately high in rural and nomadic areas, accounting for over 70% of zero-dose children compared to a national average of 44%. This rural concentration is likely exacerbated by high mobility and dispersed populations in pastoralist nomadic zones. Conflict zones also show significant inequity, with 6–15% of global zero-dose children residing in these areas. Districts characterized by poor road access, low facility density, and conflict consistently report elevated zero-dose rates. Societal determinants such as distance to services (affecting 63% of mothers) and lack of antenatal care access (affecting 73% of mothers) are strongly correlated with zero-dose status [11, 33].

#### Health system and community level determinants of non-vaccination

In Somalia, the vaccine cold chain infrastructure is organized into regional hubs to ensure effective distribution and storage. The Mogadishu hub serves Benadir, Lower Shabelle, and Middle Shabelle, covering a dense population zone. The Dhuusamareeb hub supports Galgaduud, Hiran (primarily Beletweyne), and South Mudug, catering to central regions. The Elberde hub handles cold chain operations in Bakool, while Baidoa manages logistics for the Bay region. In the southwest, Beled-Xawo is responsible for Gedo, and the Kismayo hub serves the eastern part of Lower Jubba, including Kismayo town. Additionally, the Afmadow hub covers western and southern parts of Lower Jubba, notably Afmadow and Badhadhe districts [34].

Somalia reports substantial gaps in cold chain infrastructure at Primary Healthcare Units (PHUs). In regions like Galkayo, certain health centers (e.g. Bacadwayn and Bayra) lacked functional cold storage and relied on distant regional hubs leading stockouts lasting from days up to weeks [35]. A 2025 survey in Hargeisa (Somaliland) found that over half (54.8%) of facilities demonstrated poor cold chain management, strongly correlated with staff experience, supervision, and education [36]. Earlier Effective Vaccine Management (EVM) assessments highlighted weak distributed capacity and monitoring systems, with zonal cold storage frequently falling below WHO standards [22].

Human resource constraints compound cold chain issues. Health center staff report insufficient workforce for vaccine transport and management—often fanning through transport infrastructure gaps on foot [35]. The Hargeisa survey linked poor cold chain practice to low handler experience and without strong supportive supervision [22]. EVM findings also flagged a lack of skilled technical personnel at district levels as a key bottleneck [37].

Somaliland revealed maternal literacy was significantly associated with higher vaccination uptake mothers able to read even part of a sentence were 1.61–1.89 times more likely to vaccinate their children [38]. Adult female literacy in Somalia stands at merely 22%. suggesting that low literacy contributes substantially to non-vaccination through reduced awareness and inability to interpret schedules or educational materials [38].

Among internally displaced persons (IDPs) in Mogadishu, caregivers voiced mistrust fueled by fear of side effects, misinformation, and perceived discrimination, particularly when outreach overlooked minority languages like Maay intensifying suspicion and refusal extranet. Gender norms often limit women’s autonomy; case studies show caregivers may delay or refuse vaccines if the service is delivered by male providers or without household permission [39]. Coverage among IDP populations in Mogadishu remains markedly low 49% coverage in camps versus 59% in stable urban areas. These populations face cumulative barriers: distrust, cost burdens, opportunity costs, and lower awareness contributing to higher non-vaccination rates [39]. A recent study found a 26–26.5% prevalence of missed opportunities for vaccination (MOVs) during non-immunization visits. Key factors included low caregiver knowledge, misconceptions about vaccine side effects, and logistical barriers within clinics. These systemic and knowledge gaps mean a quarter of children attending PHUs are eligible but not vaccinated [40].

#### Humanitarian access and security constraints in unliberated (inaccessible conflict-affected) districts

Humanitarian outreach in unliberated Somali districts is frequently disrupted by checkpoints, blockades, and insecurity alerts. According to ReliefWeb, in 2024 there were 243 reported access incidents—including at illegal checkpoints and armed blockades—reflecting a 35% decrease since 2023, yet significant constraints remain in al-Shabaab-controlled territories [41].These obstructions impede movement not only of humanitarian convoys but also of civilians seeking assistance. Aid agencies suspend operations during heightened threat periods or when forced to navigate forced detours through insecure zones. As Assessment Capacities Project (ACAPS) notes, “illegal checkpoints along major supply routes restrict access,” prompting costlier and riskier alternate routes such as airlifts [42].

NGOs engage in formal and informal negotiations with both state and non-state actors to gain safe passage. A notable modality is the creation of “periods of tranquility” or temporary corridors negotiated under international humanitarian law frameworks [43]. In Somalia, these negotiations often involve clan elders, local authorities, and al-Shabaab gatekeepers. The Institute of Development Studies (IDS) describes their Caafimaad Plus partners’ strategy to invest in “community reflection, operational change and negotiating with key players,” which has allowed coordinated access in otherwise inaccessible areas [42, 44].Despite the observations, such negotiations—especially with Al-Shabaab—require careful mapping of stakeholders and calibration using principled approaches grounded in humanitarian norms, while tracking the suspension or cancellation of outreach due to checkpoints, blockades, or insecurity alerts [45].

There are glaring differences when comparing access in liberated versus unliberated districts reveals stark disparities. A case study on women’s and children’s health in Somalia demonstrates that security constraints exacerbate already poor access to health services: in conflict-affected areas, community outreach is significantly reduced, and facilities are often unreachable without safe passage permissions or military escorts [46]. Survey data from the BRANCH Consortium indicates inadequate infrastructure and logistical planning, such that the nearest functional health post may lie tens of kilometers away, often beyond protected routes. Anecdotal reports within the 2020 study indicate travel times of 4–8 h one way under ideal conditions—and this assumes checkpoints are passable and escorts are available [47].

### Health implications

#### Epidemiological impact—current outbreaks and modeled future burden

Somalia is among the top 20 countries globally with the highest prevalence of zero-dose children [12, 25] and currently grappling with outbreaks of infectious diseases, including cholera, measles, dengue fever, and diphtheria, exacerbated by severe climate shocks, ongoing conflict, and a recent reduction in humanitarian funding. These challenges have led to critical gaps in vaccination coverage, further intensifying the public health crisis. Widespread displacement has left millions in precarious conditions, with limited access to clean water, sanitation, and healthcare, significantly increasing the risk of preventable diseases. Children are especially vulnerable, experiencing disproportionately high rates of severe malnutrition and disease, while cuts in aid have forced the closure of health clinics, disrupted essential services and worsening the already dire situation [25].

Somalia continues to experience annual measles outbreaks, with a major surge in 2017 (23 039 suspected cases) and a cumulative 3 509 suspected cases during weeks 1–9 of 2022 alone—81% among children under five [48]. recent retrospective analysis highlights a pooled mean CFR of 2.2% (hospital-based average 2.9%, ranging 0.9–6.0%) [49]. Age distribution data from 2011–2021 show considerable involvement of older groups (e.g., Dollo-Ado outbreak: 44.7% male, 55.3% female, with 43.7% aged ≥ 15 years) [49].These patterns align closely with the identified zero-dose communities, such as pastoralist, rural, and conflict-affected settings, as these populations typically face barriers to accessing immunization services. The findings highlight that these groups, often living in hard-to-reach areas, are disproportionately affected by low vaccination coverage, which contributes to the higher rates of zero-dose children in these regions [25].

Surveillance of acute flaccid paralysis in Somalia (2017–2024) shows consistent performance above WHO sensitivity thresholds; nonetheless, 12% of non-polio AFP cases had received no OPV doses, with 95% from South-Central regions and 60% from inaccessible districts [50, 51]. Vaccine-derived poliovirus type 2 (cVDPV2) has been detected, with 39 cases reported between 2017 and March 2024, 87% of which were concentrated in South-Central states and typically among zero-dose children (mean age 36 months; 49% female) [51].

In 2023, Somalia reported approximately 16,989 suspected cholera cases with 43 associated deaths, resulting in a case fatality rate (CFR) of 0.3%. In 2024, the country saw a cumulative total of 18,218 cases and 138 deaths, leading to a CFR of 0.8%[52]. Historical data indicate that the 2017–2018 cholera wave involved approximately 78 000 cases and 1 159 deaths (CFR ~ 1.5%) [53]. These outbreaks cluster in the same regions identified as zero-dose hotspots, exacerbated by limited access to both vaccination and WASH (water, sanitation, hygiene) infrastructure.

#### Public health consequences

Low vaccination rates in Somalia significantly strain the public health system. This has led to annual measles outbreaks—3,509 suspected cases reported in early 2022 alone—placing heavy demands on limited surveillance and emergency response capacities [48]. Likewise, between January 2017 and March 2024, 39 cases of circulating vaccine-derived poliovirus type 2 were identified across 14 regions, with 12% of children with acute flaccid paralysis never having received polio vaccines, predominantly in security-compromised areas [51]. These preventable disease outbreaks not only overwhelm scant healthcare resources but also disrupt routine services and divert funding toward emergency interventions [54]. The resulting feedback loop—where disease undercuts healthcare infrastructure, which in turn hampers vaccination efforts—is exacerbated by persistent insecurity and political instability, which obstruct campaigns in underserved regions [12]. Consequently, fragile public health functions struggle to maintain essential surveillance, routine immunization, and outbreak response in the face of recurrent epidemics and chronic underfunding.

Polio, a debilitating viral disease capable of causing permanent paralysis, continues to pose a grave public health threat in Somalia. Despite global progress in eradication efforts, Somalia has experienced vaccine-derived polio outbreaks due to persistently low immunization coverage—particularly in conflict-affected and rural areas. In 2019, Somalia reported three cases of circulating vaccine-derived poliovirus type 2 (cVDPV2), with approximately 15 children paralyzed since the outbreak's onset in 2017[55]. Contributing factors include routine bOPV (4 doses) and IPV (2 doses) coverage rates below 50% as of 2018–2019[51]. The resurgence of polio in 2019—resulting in more than 200 children across the Horn of Africa being affected—has been directly linked to immunization gaps in hard-to-reach and insecure communities [56]. Consequently, polio eradication strategies in Somalia remain critically dependent on expanding vaccination reach, particularly through intensified campaigns and enhanced access in underserved regions [57].

Pertussis (whooping cough), a highly contagious respiratory disease, poses a serious threat to infants in Somalia, where vaccination coverage remains alarmingly low. Recent data indicate that only 30–40% of children receive the full series of diphtheria–tetanus–pertussis (DTP) vaccines, leaving a majority vulnerable to infection [16]. In this context, outbreaks of pertussis are frequent, and unvaccinated infants face severe complications, including apnea, pneumonia, seizures, and even death—conditions documented globally and especially prevalent where vaccine gaps exist [58]. Somalia’s fragmented healthcare infrastructure, exacerbated by conflict and displacement, hampers routine immunization efforts, further facilitating pertussis transmission [16]. Urgent strategies—such as community outreach, mobile clinics, and integration with maternal health services—are critical to improving DTP immunization rates and protecting vulnerable infants from this preventable yet potentially fatal disease.

### Immunization strategies

Somalia faces significant immunization challenges, marked by a large population of zero-dose children [25]driven by conflict, displacement, poor healthcare infrastructure, and logistical issues. National strategies focus on high-risk groups, including children under five and pregnant women. Key interventions include strengthening the cold chain, increasing community awareness, improving data management through the HMIS, and utilizing mobile vaccination teams for remote access [59].The national Expanded Programme on Immunization (EPI) increasingly relies on roving/mobile teams that travel with cold boxes to pastoralist settlements, IDP camps, and areas temporarily inaccessible to fixed facilities. A 2024 qualitative study across Puntland, Galmudug and Jubbaland found that mobile teams were the only routine service encountered by nomadic households, yet deployment schedules were irregular and poorly micro-planned [12]. Since 2022, WHO and the Ministry of Health have paired routine vaccination with vitamin A, de-worming and COVID-19 shots during “integrated outreach days”. The catch-up campaign identified 139 350 zero-dose children and succeeded in giving a first vaccine dose to 60% of them within five months [60]. The number of health facilities offering immunizations grew from 706 in 2021 to 849 in 2022, and selected primary-care outpatient departments now function as “close-to-client” immunization points. Coverage of the first pentavalent dose (Penta-1) rose from 63% (2020) to 80% (2022), although the Penta1-to-Penta3 drop-out rate remains 15–22%[61].

Multiple studies Key informants cite insecurity, population displacement and lack of accurate denominators as the main barriers; vaccinators may travel three days to reach a settlement only to find the target population has moved on. Stock-outs, an ageing cold-chain, and under-funded supervision further undermine performance [12, 62, 63]. Piloted in Banadir in 2025, the DHIS2-based electronic Immunization Registry (eIR) registers every child, sends Somali-language SMS appointment reminders, and produces line-listing dashboards that let vaccinators spot missed doses in real time. Fourteen facilities using the system recorded a notable uptick in on-time Penta-3 completion during the first six months. National scale-up is now planned [64].

Gavi’s 2024 landscape review highlights how GIS-enabled micro-planning, tablet-based tally sheets and near-real-time dashboards reduce denominator errors and help supervisors redeploy outreach teams dynamically during outbreaks or displacement surges. In neighboring Yemen, geospatial modelling showed that adding 300 optimally located mobile sites could raise < 30-min travel access to vaccines by 7% of the under-5 population; Somalia’s programme is adapting the same algorithm to IDP settlements around Kismayo and Baidoa [63, 65].

The ALMANACH app is a digital pediatric care tool that aids healthcare providers in managing childhood illnesses using algorithms to guide consultations and recommend examinations, referrals, diagnostic tests, and age/weight-based medication dosages [66].The ALMANACH app, rolled out in seven south-central health facilities, cut inappropriate antibiotic use for childhood illnesses by 72% and raised adherence to follow-up counselling to 94%. Besides improving overall quality of pediatrics care, ALMANACH pings vaccinators when a child’s record shows missing doses, creating an extra safety-net for zero-dose identification [67].Digital registries and data quality. Paper-based recording dominates, hampering defaulter tracing—despite pilots of an electronic immunization register cited in the national multiyear plan [68].Experience from the 2022–2023 COVID-19 drive demonstrated that redeploying polio front-line workers, using micro-plans at district level, and co-locating adult and child vaccination posts can boost routine coverage even in fragile settings. These levers are now being formalized into the national “Reach Every Settlement” strategy [62]. Evidence from Somali case-studies stresses the value of involving women’s groups, religious leaders and school networks in social mobilization, and of integrating livestock vaccination campaigns in nomadic areas to share logistics costs and build trust **(**Table 1) [12, 63].

**Table 1:** Key challenges to reaching zero-dose children in inaccessible, conflict-affected areas of Somalia

Theme/area	Specific challenges in inaccessible/conflict-affected Somalia	Evidence signals from your results	Impact	IA2030-aligned response (Brief)
Epidemiological burden	Very high zero-dose prevalence; clustered hotspots	60% zero-dose nationally; district peaks up to ~ 62% (Lower Juba/Bay); 44% DPT1 zero-dose	Recurrent measles/cVDPV2/cholera	Target zero-dose mapping; equity micro-plans per district
Geography & mobility	Rural/nomadic dispersion; IDP movements	74% of zero-dose in rural/nomadic; frequent population shifts	Missed sessions; high drop-outs	Mobile/outreach teams with dynamic micro-plans; GIS routing
Humanitarian access & security	Checkpoints, blockades, insecurity in unliberated areas	~ 243 access incidents (2024); periodic suspensions	Interrupted outreach & supervision	Negotiated access corridors; local mediators; “days of tranquillity”
Supply chain & cold chain	Aged equipment; gaps at PHUs; stock-outs	> 50% poor cold-chain practice (Hargeisa survey); hub-dependent transport	Potency loss; service interruptions	Solar direct-drive fridges; passive carriers; buffer stocks
Workforce & service delivery	Insufficient vaccinators/supervision; irregular outreach	Irregular mobile rounds; long travel times; limited supervision	Inconsistent availability; quality issues	Fund/schedule regular outreach; supportive supervision loops
Data & surveillance	Weak denominators; paper registers; poor defaulter tracing	Penta1 → Penta3 drop-out 15–22%; pilots show eIR improves timeliness	Invisible missed children; slow corrective action	Scale DHIS2-linked eIR + SMS; integrate AEFI & VPD dashboards
Demand & trust	Mistrust, misinformation, gender norms, language barriers	Low caregiver knowledge; refusal fears; IDP language exclusion	Low uptake even when supply exists	Female CHWs; tailored SBC; community co-design materials
Governance & financing	Heavy donor reliance; fragmented coordination	Govt ~ 0.3% of vaccine costs (2014); parallel ICCs; stock-outs	Fragile continuity; duplication/waste	Immunization Trust Fund; unified ICC dashboard & accountability
WASH & PHC linkages	Poor WASH, weak PHC functionality in hotspots	Cholera surges (high CFR); limited PHC access	Higher VPD transmission & mortality	Bundle WASH + EPI in outreach; PHC revitalization in hotspots
Sustainability & shocks	Aid cuts risk post-2025; climate shocks	Reported funding uncertainty; drought/flood cycles	Programme stop-start; coverage backsliding	Medium-term fiscal roadmap; emergency buffers (CERF/CBPF)

### Policy and governance

Somalia’s Expanded Programme on Immunization (EPI) is guided by the 2021–2025 national EPI policy, which the Federal Ministry of Health (FMoH) co-developed with WHO and UNICEF. The policy embeds immunization in the Essential Package of Health Services and mandates five routine contacts before a child’s first birthday. It has extended service points to 607 facilities across 117 of 123 districts and relies on NGO-managed clinics in insecure zones [68]. Following a joint appraisal of local priorities, Somalia introduced the pneumococcal conjugate vaccine (PCV) and rotavirus vaccine nationwide in April 2025, marking its first new antigens since 2013 [69]. At global level, Somalia is a signatory to Immunization Agenda 2030 (IA2030), which calls for 90% DTP3 and MCV2 coverage and a 50% reduction in zero-dose children by 2030. Yet the 2024 IA2030 progress report shows that Somalia’s DTP3 coverage remains below 60%, far from the 90% goal [70, 71].

Domestic legislation and financing to Somalia lacks an immunization act that earmarks domestic funds; donor contributions still account for > 80% of vaccine costs, creating year-to-year uncertainty. Zero-dose children. UNICEF’s 2023 zero-dose analysis places Somalia among six countries with DTP1 coverage ≤ 50%, underscoring persistent equity gaps in hard-to-reach districts [72].

A 2025 peer-reviewed review highlights chronic under-investment: the FMoH’s per-capita health allocation is < US$ 15, well below the US$ 34 WHO benchmark for essential services; irregular donor disbursements force stop-start outreach rounds and salary delays for vaccinators [73]. Two Inter-Agency Coordination Committees (ICCs) exist (Mogadishu and Hargeisa), but parallel reporting lines through polio infrastructure and multiple NGO partners produce fragmented micro-plans and duplicate cold-chain inventories. WHO reports continuing efforts to harmonise these via joint campaign planning, yet full integration into a single national EPI dashboard is pending [68].

Comparative evidence from northwest Syria shows that hybrid governance models—blending local health directorates with UN oversight—can sustain cold-chain integrity under similar conditions, suggesting transferrable lessons for Somalia [74]. Only 43% of facilities submitted complete EPI monthly reports in 2024, and external audits found inconsistencies between child registers and the DHIS2 platform. Limited public disclosure of ICC minutes constrains community oversight and fuels mistrust; a determinant of under-vaccination identified in recent Somali household surveys [16].

### Community engagement

Community engagement is increasingly recognized as a decisive lever for shifting vaccine-related attitudes in fragile settings such as Somalia. A 2022 cross-sectional survey of 1 281 Somali health-care workers showed that uptake of COVID-19 vaccine was strongly predicted by trust in local information channels and by perceptions of peer acceptance, underscoring the social nature of hesitancy [75]. One way communities have tackled misinformation is by co-creating culturally tailored educational tools. A Somali refugee community in the United States, for example, worked with researchers to design a Somali-language virtual-reality (VR) experience on childhood immunizations; iterative community co-design markedly improved intention to vaccinate among participants, illustrating how empowerment can shift behaviors [76].

Recent immunization drives in Somalia highlight the benefits of empowering communities to take a leading role in the process, resulting in more successful outcomes. During the April–May 2025 integrated measles, pneumococcal conjugate vaccine (PCV) and novel oral polio vaccine (nOPV2) campaign, mothers, clan elders and youth volunteers helped map households, escort mobile teams and run on-site feedback sessions—contributing to > 3 million children reached with measles vaccine and 2.6 million with PCV [77]. Somalia’s adult COVID-19 rollout likewise adopted “micro-planning” with district committees and civil-society groups. This community-led targeting raised adult coverage from 13% in mid-2022 to 42% by December 2022, while simultaneously vaccinating 84 600 previously zero-dose children during bundled outreach [62]. Globally, the Global Polio Eradication Initiative (GPEI) offers additional lessons: in conflict-affected zones it has relied on locally recruited social mobilisers and “convenience posts” agreed with community leaders, achieving repeated rounds of supplementary immunizations even where fixed facilities were inaccessible [78].

Evidence from 219 studies across humanitarian settings shows that CHWs can sustain essential preventive services—including vaccination—when formal systems falter, provided they are selected by, and accountable to, their own communities [79]. A 2023 rapid global review identified at least 20 countries where trained CHWs legally administer vaccines, reporting gains in coverage (up to 30 percentage-points in hard-to-reach areas) and improved timeliness when the cadre is integrated into the supply chain and supervision system [79].

In Somalia, CHWs trained as “vaccination champions” were central to the 2025 integrated campaign—conducting door-to-door social-mobilizations, registering children via a mobile app and giving nOPV2 drops in nomadic settlements steps highlighted by WHO as instrumental in closing rural–urban equity gaps [62, 77]. Their status as trusted neighbors also helps mitigate security-related access barriers that frequently limit outside teams.

## Future directions and recommendations

### Strategic recommendations

#### Reach and sustain zero-dose reduction through people-centered micro-planning

Somalia’s 44 % prevalence of “zero-dose” children aged 12–23 months highlights an urgent coverage gap [25]. Facility-level micro-plans—developed with community leaders, mapped outreach sites and budgeted logistics—have already been adopted in the national a comprehensive multi-year plan for immunization (cMYP) and shown to improve equity [22, 72]. Scaling this approach nationwide, with a focus on nomadic and peri-urban settlements, would directly advance IA2030 Strategic Priority 3 (leave no one behind) [28].

#### Modernize surveillance and data use

IA2030 calls for comprehensive vaccine-preventable disease surveillance supported by strong, reliable laboratory networks [28]. Building an electronic immunizations registry that integrates DHIS2 with GIS mapping will allow near-real-time defaulter tracing, while linking Adverse Events Following Immunization (AEFI) reporting to national pharmacovigilance will strengthen confidence. Recent Somalia experience with adding rotavirus and PCV vaccines shows that coverage accelerates when surveillance data drive rapid corrective action [59].

#### Embed resilience for health-security shocks

IA2030 Strategic Priority 5 stresses service continuity in humanitarian settings [28]. Ensuring a climate-resilient cold-chain (solar direct-drive refrigerators, passive containers) and pre-positioned supplies in each Federal Member State will mitigate disruption. Somalia’s 2014 CERF-funded measles response demonstrated how pooled emergency grants can fill immediate gaps while national systems mobilize longer-term resources [80].

### Sustainability considerations

#### Diversify and align financing

Government contributions to routine immunizations were < 1% of programme costs in 2014, with heavy donor reliance [22]. A progressive fiscal roadmap earmarking 0.3% of general health expenditure for vaccines by 2027 and 0.5% by 2030—would align with IA2030 Strategic Priority 6 on sustainable financing [28]. Complementary mechanisms include: Country-Based Pooled Funds (CBPF/CERF) to underwrite rapid outbreak response while domestic funds build up [80]. Immunization Trust Accounts are managed jointly by the Ministry of Health and the Ministry of Finance, ensuring transparent disbursement of funds. This structure facilitates co-financing with Gavi transition grants. Additionally, performance-based conditional grants are allocated from the federal government to state ministries, linked to quarterly coverage targets. This approach has helped reduce the projected funding gap of US $32 million for the 2016–2020 period [22].

## Strengthen governance and accountability

A multi-sectoral National Immunization Coordination Committee (NICC) chaired at ministerial level should use quarterly scorecards to track IA2030 indicators and publish results publicly. Incorporating civil-society observers and community representatives (especially women’s groups) into NICC meetings institutionalizes the participatory ethos of IA2030^1^ and the pro-equity planning lessons from UNICEF micro-plans [11].

## Discussion

This narrative review highlights that Somalia is experiencing one of the most severe zero-dose crises globally, with over 60% of children remaining completely unvaccinated in several conflict-affected regions, including the Lower Juba and Bay districts [11, 25]. Spatial analyses illustrate tightly clustered “cold spots” that coincide with zones of chronic insecurity, weak road infrastructure, and high population mobility—features that collectively impede the delivery of even the first contact in the immunizations schedule. Although national coverage for the first pentavalent dose improved from 63% in 2020 to 80% in 2022, the persisting 15–22% Penta-1–to-Penta-3 drop-out highlights systemic fragilities that continue to generate large cohorts of zero-dose and under-immunized children [61].

The Somali pattern mirrors broader evidence from fragile and conflict-affected states (FCAS), where children living in insecure or remote settings are 1.5–2 times more likely to be zero-dose than their urban peers [33]. Nevertheless, two distinctive characteristics intensify Somalia’s predicament. First, militant control effectively severs routine supervision and resupply to many primary-health-care units (PHUs); in 2024 alone, 243 access incidents—checkpoint blockades, armed attacks, and convoy detentions—were recorded, curtailing outreach to large swathes of South-Central Somalia [41, 46]. Second, Somalia’s EPI remains 99% donor-financed, with government spending covering barely 0.3% of vaccine costs as recently as 2014 [22]. Dependence on volatile external funding magnifies the shock of every fiscal delay or aid suspension, as observed during 2023–2024 supply-chain interruptions that triggered stock-outs of pentavalent and measles-rubella vaccines in Baidoa and Kismayo [73].

Our findings reinforce the epidemiological consequences of these gaps. Persistent zero-dose pockets sustain endemic circulation of measles, cholera, and vaccine-derived poliovirus type 2, each outbreak siphoning scarce surveillance and clinical resources from an already overstretched health system [48, 51, 81]. Modelling studies in comparable low-coverage settings suggest that increasing routine DTP3 and MCV1 coverage by 20 percentage-points could avert tens of thousands of under-five deaths within 5 years [82]. Extrapolated to Somalia, achieving even the interim IA2030 target of 70% DTP3 in every district would markedly reduce the case-fatality burden while bolstering regional health-security objectives.

Several promising strategies documented in the results warrant accelerated scale-up. Community-led micro-planning—where clan elders, women’s groups, and youth volunteers map settlements and co-design campaign schedules—has repeatedly improved last-mile reach during integrated measles-polio-PCV rounds, vaccinating more than three million children in April–May 2025 alone [77].Likewise, early pilots of a DHIS2-linked electronic immunizations registry (eIR) in Banadir demonstrated real-time defaulter tracing and improved on-time Penta-3 completion [64]. These digital innovations address two chronic bottlenecks—denominator uncertainty and weak follow-up—yet their sustainability will hinge on stable electricity, data connectivity, and long-term financing for server hosting and user support.

Notwithstanding these gains, substantial policy and governance gaps persist. The dual Inter-Agency Coordination Committees in Mogadishu and Hargeisa still operate parallel cold-chain inventories and micro-planning cycles, complicating national oversight [68]. Moreover, immunization remains outside Somalia’s draft Public Health Bill; without legislated budget lines, domestic co-financing targets set by Gavi’s fragility policy risk slipping further. Experience from Rwanda and Ethiopia shows that codifying an “Immunisation Fund” within health-sector legislation can protect allocations during fiscal downturns and facilitate pooled procurement agreements that lower unit vaccine prices. Somalia’s Ministry of Finance and Federal Member States could therefore adapt such a model, earmarking at least 0.5% of general health expenditure to vaccines by 2030, as proposed in this review’s fiscal roadmap.

A further recurrent theme is community trust. Qualitative studies among internally displaced persons in Mogadishu expose deep-rooted concerns over vaccine side-effects, linguistic exclusion, and gender norms that restrict women’s autonomy to seek services when only male vaccinators are present [39]. These social barriers cannot be resolved by supply-side fixes alone. Evidence from the Global Polio Eradication Initiative underscores the effectiveness of locally recruited, female community health workers (CHWs) who combine household counselling with vaccine delivery, achieving up to 30-percentage-point coverage gains in hard-to-reach settlements [79]. Embedding a formally recognized CHW cadre within Somalia’s Essential Package of Health Services—and ensuring they are remunerated, supervised, and supplied—would institutionalize this successful but presently ad-hoc approach.

This review has limitations. First, the heterogeneous quality of surveillance and household survey data—especially in insecure districts—means that zero-dose prevalence may be under- or over-estimated. Second, grey literature and NGO reports, while indispensable in data-poor settings, are often not peer-reviewed, potentially introducing bias. Finally, the rapidly evolving security landscape implies that access constraints documented in 2024 may have shifted by the time of writing; periodic re-mapping is essential.

Strategic Recommendations and Future Directions:

1. Expand people-centered micro-planning. To ensure that zero-dose children in rural, nomadic, and conflict-affected areas of Somalia are vaccinated, micro-planning strategies involving local community leaders, clan elders, and women’s groups are crucial. These plans should prioritize nomadic and peri-urban populations, ensure regular outreach visits, and tailor vaccination schedules to the needs of specific regions. By scaling these approaches nationwide, Somalia can address existing gaps, particularly in high-risk zones, and work towards achieving the Immunization Agenda 2030 (IA2030) goal of "leaving no one behind."
2. Enhance surveillance and data utilization. Building an integrated electronic immunization registry (eIR) that connects with the DHIS2 system will facilitate real-time tracking of vaccination coverage and missed doses. This will help in identifying zero-dose children quickly and efficiently, particularly in areas with weak denominators. Additionally, aligning the eIR with adverse event monitoring (AEFI) will strengthen surveillance, improve immunization confidence, and allow for corrective actions when necessary. A strong data backbone will also help identify emerging trends, enabling timely interventions.
3. Strengthen health-system resilience for Health-Security Shocks. Given the climate and security challenges that Somalia faces, building resilience into the vaccination supply chain is essential. This includes using solar direct-drive refrigerators and pre-positioned passive containers to ensure the continuity of vaccine distribution, even during climatic or security disruptions. These steps will mitigate the risks of vaccine potency loss and stock-outs in fragile environments, aligning with IA2030’s commitment to service continuity in humanitarian settings.
4. Diversify and align financing for immunization. Somalia’s heavy reliance on donor funding, which accounted for over 99% of its vaccine costs in 2014, leaves its immunization program vulnerable to fluctuations in international aid. To secure long-term sustainability, the Somali government should earmark a portion of its general health expenditure for immunization, with a clear fiscal roadmap to gradually increase domestic co-financing. A progressive target of 0.5% of health expenditure by 2030 should be set, alongside establishing an Immunization Trust Fund to enable transparency, efficiency, and co-financing from both the government and international partners.
5. Formalize Community Health Worker (CHW) Cadres A key barrier to vaccination in conflict zones and remote areas is community trust, especially among internally displaced persons (IDPs) and nomadic populations. Empowering female community health workers (CHWs) and Field Epidemiology Training Program (FETP), particularly those who are trusted members of the community, can bridge these gaps. CHWs can engage in social mobilization, address vaccine hesitancy, and ensure children receive vaccines even when mobile teams are unavailable. Establishing a formal, remunerated CHW system within the Essential Package of Health Services will strengthen outreach efforts and improve overall vaccination coverage.
6. Strengthen governance and accountability. The success of immunization efforts depends on cohesive governance and effective coordination between national and regional bodies. Establishing a unified national immunization dashboard that consolidates data from multiple sources (e.g., polio infrastructure, NGOs, and government facilities) will help streamline planning and prevent inefficiencies. Additionally, a multi-sectoral National Immunization Coordination Committee (NICC) should regularly assess progress, track IA2030 indicators, and involve civil-society organizations in the decision-making process to ensure transparency and community involvement.
7. Integrate WASH and PHC systems with immunization programs. Immunization efforts in Somalia will be more effective if they are integrated with water, sanitation, and hygiene (WASH) services. The high rates of cholera and other water-borne diseases in zero-dose hotspots underline the need for simultaneous improvements in WASH infrastructure and primary healthcare (PHC) services. By bundling WASH interventions with immunization outreach, the risk of disease transmission can be reduced, leading to healthier communities and improved vaccine uptake.

## Conclusion

Somalia faces a severe and persistent challenge in addressing the high prevalence of zero-dose children, particularly in conflict-affected, inaccessible regions. Despite some national gains in immunization coverage, significant disparities remain, especially in rural, nomadic, and militant-controlled areas. The main drivers of these gaps include insecurity, inadequate healthcare infrastructure, logistical challenges, and socio-cultural barriers such as mistrust and gender norms.

To achieve the Immunization Agenda 2030 (IA2030) goals of universal vaccination and "leaving no one behind," urgent action is required. This includes scaling up people-centered micro-planning, improving surveillance and data systems, enhancing health-system resilience to shocks, and ensuring sustainable financing through increased domestic contributions. Empowering local communities, particularly women and community health workers (CHWs), is essential for overcoming barriers to vaccination and ensuring that outreach reaches the most marginalized populations.

By embedding these strategies into the national health framework, Somalia can make significant strides in reducing zero-dose rates, improving immunization equity, and building a resilient healthcare system capable of withstanding both political instability and environmental shocks. The collective efforts of the government, international partners, and local communities will be crucial to achieving these objectives and securing a healthier future for Somalia’s children.

## Data Availability

Not applicable.
